# Optimization of Printing Parameters to Enhance Tensile Properties of ABS and Nylon Produced by Fused Filament Fabrication

**DOI:** 10.3390/polym15143043

**Published:** 2023-07-14

**Authors:** Andrei Yankin, Yerassyl Alipov, Ali Temirgali, Gaini Serik, Saniya Danenova, Didier Talamona, Asma Perveen

**Affiliations:** Department of Mechanical and Aerospace Engineering, School of Engineering and Digital Sciences, Nazarbayev University, Astana 010000, Kazakhstan; andrei.iankin@nu.edu.kz (A.Y.); yerassyl.alipov@alumni.nu.edu.kz (Y.A.); ali.temirgali@alumni.nu.edu.kz (A.T.); gaini.serik@alumni.nu.edu.kz (G.S.); saniya.danenova@alumni.nu.edu.kz (S.D.); didier.talamona@nu.edu.kz (D.T.)

**Keywords:** FFF, FDM, ABS, nylon, tensile test, parametric study

## Abstract

This study aimed to identify the optimum printing parameters for the fused filament fabrication (FFF) of acrylonitrile butadiene styrene (ABS) and polyamide (nylon), to improve strength properties. For this purpose, the methodology of the paper involves an experimental study that used Taguchi’s method to identify the effects of the infill pattern, infill density, and printing speed on the mechanical properties of the materials. ABS and nylon plastic parts were tested in tension to failure. Based on the results of the tensile tests, it was found that ABS material produced the highest ultimate tensile strength when printed using a tri-hexagonal infill pattern, 100% infill density, and a printing speed of 65 mm/s. On the other hand, nylon material exhibited a better performance when printed using an octet geometric structure, with identical other parameters.

## 1. Introduction

Additive manufacturing (AM) refers to a relatively novel approach to a rapid prototyping technique that enables the creation of an object, layer by layer, with the help of a plastic material or metal powder [1]. The capabilities of 3D-printing technologies are wide enough to produce an object of almost any shape, including shapes unlikely to be achieved via conventional methods, and avoiding the use of massive machines and high levels of manpower.

**Figure 1:**
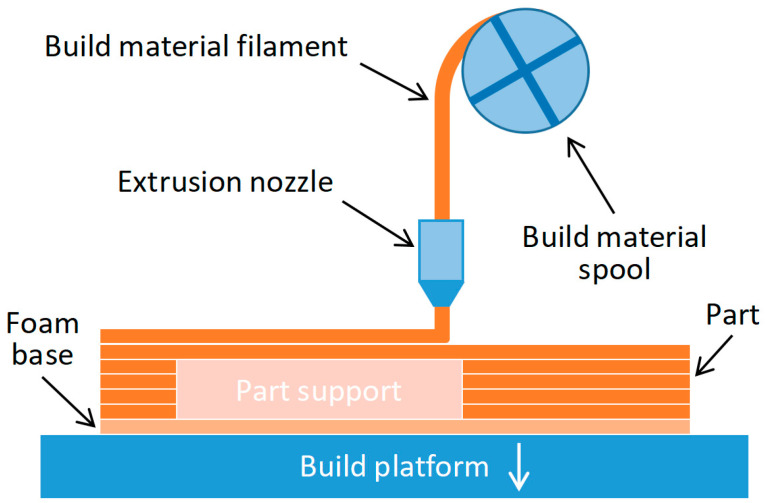
Fused filament fabrication process schematic.

Fused filament fabrication (FFF) is the most widely used and recognized AM technique (Figure 1), primarily due to its affordability and accessibility as a desktop printer, compared to other methods. FFF printing is particularly valuable in producing cost-effective parts quickly, creating rigid models, and constructing prototypes for validation purposes [2]. As a result, the range of applications of 3D-printed parts using FFF technology and thermoplastic materials is rapidly expanding. The automotive, aerospace, medical, industrial, manufacturing, and architecture industries, among others, are increasingly incorporating FFF technology into their processes [2,3].

In FFF, a thermoplastic filament is melted and used to construct the cross-sectional geometry of an object on a build platform. Various materials can be utilized in FFF, including polycarbonate (PC), polylactic acid (PLA), acrylonitrile butadiene styrene (ABS), polyamide (nylon), and more [4,5,6,7]. The printing process involves heating the thermoplastic polymer filament to its viscous point, and then extruding it through a nozzle, in a layer-by-layer fashion, onto a glass plate. The layers adhere to one another through the molecular interaction between the extruded molten filament and the solidified layer. This thermoplastic behavior allows for the fusion of layers, and facilitates the transition from a molten state to a solid state, as the temperature decreases.

Despite the numerous advantages of FFF printing compared to conventional manufacturing methods, certain limitations need to be addressed. The wide range of limitations includes the lower mechanical properties, and inferior surface quality, of the printed parts. One of the key challenges is to address the formation of voids between the layers in printed parts [8]. This issue arises from the relatively weak interaction between the extruded layer and the solidified part [9]. Consequently, further research is necessary to investigate and enhance the mechanical properties of the final printed object.

The process parameters impacting FFF printing include the infill density, infill patterns (internal geometric structure), extrusion temperature, nozzle diameter, layer thickness, raster angle, build orientation, printing speed, etc. [7,10,11]. The parameters identified as the most influential, based on previous studies, are selected, to determine the mechanical properties of FFF printed parts.

The infill density refers to the amount of material filling the internal structure of the printed part, which can vary from primarily hollow (0%) to mostly solid (100%), depending on the design and requirements. Extensive research has shown that infill density is one of the primary factors that significantly affects the printed part’s strength. Studies have demonstrated that increasing the infill density enhances the strength of printed materials such as ABS, PLA, and nylon [12,13,14,15].

In addition to the infill density, the internal geometric structure of the FFF printed parts is also crucial for achieving the desired mechanical properties. The internal structure defines how the infilled filaments interact when the part is subjected to loading. Different internal structure shapes, such as triangular, gyroid, cubic, and more, can be utilized. The effect of infill patterns on mechanical properties has been investigated in studies [16,17]. It is important to note that each infill pattern may yield different results for specific mechanical properties. While a particular pattern might be effective for enhancing tensile or compressive properties, it may not perform as well for components subjected to other loads. Therefore, careful consideration and evaluation of the intended application and desired mechanical behavior are necessary when selecting the appropriate infill pattern for FFF printed parts.

The printing speed, in FFF, refers to the rate at which the nozzle and other movable parts of a 3D printer move, in relation to the stationary components. This parameter aims to strike a balance between the printing time and the quality of the printed parts. When the printing speed is set too high, it can result in a weak interaction between the extruded layer and the solidified part. Several studies [18,19,20] have demonstrated that increasing the printing speed negatively affects the tensile strength of the FFF parts. It is essential to consider the printing speed carefully, to ensure optimal printing outcomes. Finding the right balance between the printing speed and the desired mechanical properties of the printed part is crucial for achieving satisfactory results in FFF printing.

Therefore, as indicated in the literature mentioned earlier [12,13,14,15,16,17,18,19,20], the infill density, infill pattern, and printing speed play a crucial role in determining the tensile strength of 3D-printed parts. By meticulously selecting and effectively controlling these key process parameters, researchers and manufacturers can optimize the mechanical properties and overall performance of parts produced using the fused filament fabrication (FFF) technique.

As mentioned earlier, FFF technology utilizes thermoplastic materials with high strength, such as ABS and nylon. Nylon, known for its excellent impact strength, stress resistance, high tensile and flexural strength, thermal stability, and cost-effectiveness, is currently used in automotive parts [21] and medical components [22]. However, ABS is highly resistant to heat and chemicals, and is suitable for machining. Its affordability also contributes to its popularity as a material choice. ABS is applied in the automotive, household-goods, electronics, and medical-application industries, among others. In addition, FFF parts can be reinforced to enhance their mechanical and physical properties [23,24,25]. One commonly used reinforcing fiber is carbon fiber, which exhibits progressive deformation behavior, and is widely employed in strengthening polymers.

The literature review summary for ABS and nylon is presented in Table 1. Zhang H. [26] examined the effect of the printing orientation on the tensile properties of ABS. Tensile, compression, and three-point bending tests were conducted, using FFF with an ABS filament [27]. Lay M. et al. [28] suggested a relatively weaker FFF specimen when comparing the mechanical performance of ABS and nylon 6 fabricated through FFF, and through conventional injection molding. Shabana R. et al. [29] investigated the mechanical characteristics of 3D-printed ABS and PLA, concluding that the PLA showed a superior ultimate tensile strength. Kannan S. et al. [30] compared the mechanical properties of ABS, PC, and PC-ABS, using the same process parameters. The PLA and ABS were subjected to tensile and flexural tests [31], with the PLA exhibiting 7–9% higher strength. Panes A. et al. [32] also found that PLA outperformed ABS in terms of mechanical performance, considering the layer height, infill density, and layer orientation. Algarni M. et al. [33] applied ANOVA to evaluate the effects of various factors on the mechanical properties of PLA, ABS, PEEK, and PETG. The effects of the raster angles, layer height, and infill density on ABS were investigated [34]. The study in [35] explored the combined effects of different infill patterns, infill densities, and layer thicknesses.

The effects of the melting temperature and infill orientations on nylon and ABS were investigated in [36], revealing significant alterations in the sample attributes based on the nozzle temperature and infill line orientations. Terekhina et al. [37] studied the impact of internal filling on the strength characteristics of nylon, finding a significant increase in strength when the volume fraction of the infill structure was above 60%. The influence of various FFF process parameters on the tensile strength and the modulus of elasticity of 3D-printed nylon 12 was examined using Taguchi’s L18 orthogonal array in [38]. The effects of the printing speed, layer height, and infill density on the nylon printed parts were explored in [13]. Moradi M. et al. [39] investigated the effects of the infill percentage, layer thickness, and number of contours, and their interactions, on the mechanical properties of nylon 12, using a design-of-experiment method. The study in [40] focused on the influence of the air gap, raster angle, and build orientation on the flexural strength of FFF-manufactured nylon 12 parts, highlighting the significant impact of the air gap and raster angle. In [41], the process parameters were optimized for the FFF process, using Taguchi’s L9 orthogonal array for ABS and nylon.

**Table 1:** The literature review summary.

Material	Printed Parameters	Results	Ref
ABS	- nozzle temperature: 220–230 °C - nozzle diameter: 0.5 mm - printing speed: 30 mm/s - layer height: 0.1 mm - infill density: 100%	Young’s modulus for 0, 45, and 90 deg of printing orientation are 1.81 GPa, 1.80 GPa, and 1.78 GPa, respectively, while the ultimate strength values are 22.4, 20.7, and 19.0 MPa, respectively.	[26]
ABS	- melting temperature: 220 °C - nozzle diameter: 0.5 mm - printing speed: 50 mm/s - layer height: 0.15 mm - infill density: 50%	Elasticity modulus: 0.65 GPa, strength: 21.68 MPa.	[27]
ABS Nylon	- melt. temp.: 230 °C (ABS), 250 °C (nylon) - nozzle diameter: 0.4 mm - printing speed: 60 mm/s - layer height: 0.15 mm - infill density: 100%	ABS: elasticity modulus: 1.3 GPa, ultimate strength: 43 MPa. Nylon: elasticity modulus: 1.45 GPa, ultimate strength: 49 MPa.	[28]
ABS	- melting temperature: 250 °C - printing speed: 35–55 mm/s - layer height: 0.1 mm - infill density: 100% - raster orientation: ±45 deg	Elasticity modulus: 1.3 GPa, ultimate strength: 34.5 MPa.	[30]
ABS	- raster angle: 0–90 deg - layer thickness: 0.1–0.3 mm - infill percentage: 60–100% - printing speed: 20–40 mm/s	The infill density is the key process variable that affects ABS strength and elastic modulus, the layer thickness is the second, the raster angle is the third, and the printing speed is the last.	[33]
ABS	- layer height: 0.35, 0.4, and 0.5 mm - infill density: 40, 60, and 80% - raster angles: 45, 55,and 65 deg	The optimum parameters are an 80% infill percentage, 0.5 mm layer thickness, and 65° raster angle. Tensile strength 31.57 MPa, elastic modulus 0.77 GPa, yield strength 19.95 MPa.	[34]
ABS	- layer height: 0.1, 0.2, and 0.3 mm - infill density: 75, 80, and 85% - infill patterns: line, triang., and concent.	The best results were obtained with a concentric infill pattern, along with an 80% infill density and 100 μm layer thickness. Tensile strength 38.95 MPa, yield strength 30.07 MPa.	[35]
Nylon	- melting temperature: 240 °C - nozzle diameter: 0.3 mm - printing speed: 40 mm/s - layer height: 0.15 mm - infill density: 20, 40, 60, 80, 100%	At the infill density of 20–40%, neighboring tracks of the same layer do not touch each other. When it is increased to 60%, the parallel tracks make contact, which leads to the formation of a continuous layer, and increases the strength of the entire sample.	[37]
Nylon	- infill density: 10–100% - layer thickness: 0.178–0.33 mm - no. of contours: 1–5 - raster pattern: sparse, double - raster width: 0.457–0.71 mm - no. of shells: 2–4 - raster orientation: 0–45 deg	The infill density is the most significant parameter. The number of contours is the second. The layer thickness ranks third. The number of shells is the fourth. The raster pattern has the lowest significance. The first four parameters control about 80% of the response value.	[38]
Nylon	- printing speed: 60–70 mm/s - layer height: 0.1–0.3 mm - infill density: 50–100%	The infill density has the highest contributing factor to mechanical characteristics, the layer thickness is second, and the printing speed is the last.	[13]
Nylon	- layer thickness: 0.15–0.35 mm - infill percentage: 15–55% - number of contours: 2–6	The layer thickness is the significant primary variable for all responses.	[39]
ABS Nylon	- layer thickness: 0.1–0.3 mm - orientation angle: 0–30 deg - shell thickness: 0.4–1.2 mm	For ABS, the tensile strength is max. for 0.2 mm layer thick, a 150 orient. angle, and 1.2 mm shell thickness. For nylon, the tensile strength is max. for 0.1 mm layer thick, a 300 orient. angle, and 1.2 mm shell thickness.	[40]

Although numerous studies have examined the mechanical behavior of FFF-printed parts, most of them concentrate on modifying one or two process parameters. Additionally, a significant portion of the research focuses on the commonly used plastics in FFF; namely, ABS and PLA. On the other hand, nylon, which is less frequently utilized, is applied in the production of gears and friction pairs that undergo cyclic loads, meaning that it is more often subjected to fatigue testing. Consequently, there is a relative scarcity of data in the literature regarding the mechanical properties of nylon, with only a limited number of reports available on its tensile strength. However, the prospects for utilizing nylon as a matrix for composite materials are growing, because of its high compatibility with biodegradable natural fibers. This can significantly expand the scope of the material [35,37,41].

The previous research of the authors focused on the fatigue performance of FDM printed parts made of ABS and nylon [42]. This current study focused on an advanced parametric analysis of these materials, using experimental testing. The objective was to identify the optimal parameter configurations that could enhance the mechanical properties of FFF-printed ABS and nylon. Tensile testing was employed, to characterize the mechanical properties of the printed parts. The study investigated various factors that influence the performance of 3D-printed parts, including the internal geometric structure (infill pattern), printing speed, and infill density.

## 2. Materials and Methods

**Figure 2:**
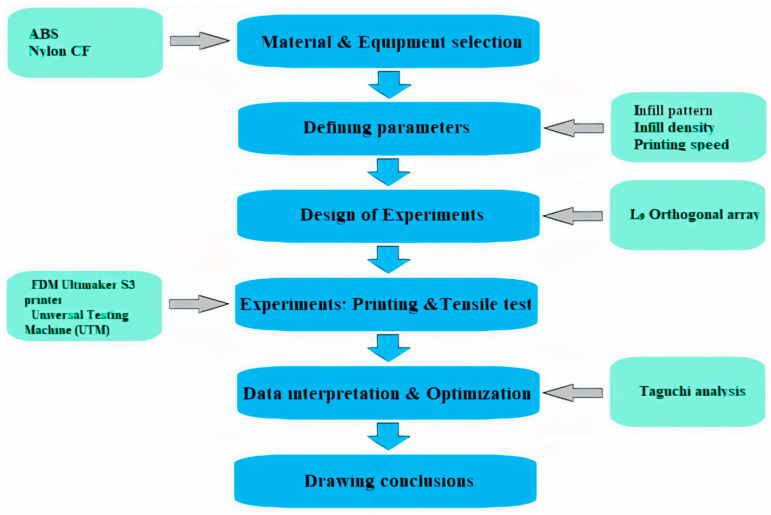
Methodology steps.

Figure 2 shows the methodological steps that were used during this study. It starts with the selection of filament types, and continues with defining the parameters for the tensile test. Both ABS and PA6 (nylon) filaments, manufactured by UltiMaker (Utrecht, Netherlands), were selected to be examined in an experimental tensile test. For nylon, the impact strength was 14 kJ/m^2^, the thermal resistance was 89 °C, and the filament diameter was 2.85 mm. For ABS, the impact strength was 14 kJ/m^2^, the thermal resistance was 87 °C, and the filament diameter was 2.85 mm. Additional information can be found on the website of the manufacturer. To study the tensile strength of the FFF-printed materials, three parameters were chosen: the infill density, infill pattern, and printing speed.

The L9 orthogonal array for Taguchi optimization was implemented to build the design of experiments (DOE). During the experimental part of the work, samples were printed according to the DOE. Then, tensile testing was conducted with the samples, to study their Young’s modulus, ultimate tensile strength, and yield strength at different parameter configurations. The test results were analyzed using Taguchi analysis. After that, conclusions were drawn on the best configurations of the printing parameters.

### 2.1. Specimen Design

**Figure 3:**
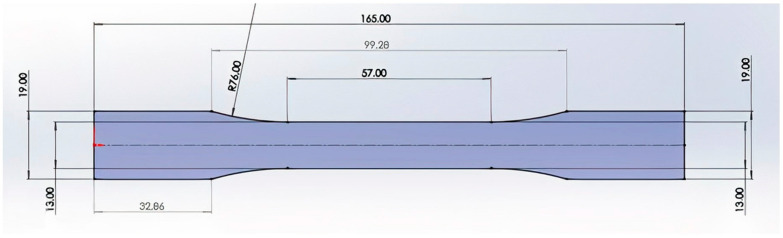
Specimen for the tensile test.

For the tensile test, the specimen was designed according to the ASTM D638 Type I standard (165.0 mm × 19.0 mm × 3.2 mm), as shown in Figure 3. The model was fabricated in SolidWorks software, and exported as an STL file.

### 2.2. Design of Experiment

The Taguchi method, developed by Genichi Taguchi, is a statistical approach that aims to design experiments, and optimize processes, to enhance product quality, reduce variability, and minimize the influence of noise factors on product performance [43]. Utilizing orthogonal arrays as the experimental design ensures a minimal number of tests, while encompassing all relevant factors and their levels. The method primarily focuses on determining the optimal parameter settings that mitigate the impact of noise factors on product performance. Additionally, it emphasizes the design of processes that can accommodate variations in product performance, thus promoting consistent and reliable results. With its systematic and efficient approach to experiment design and parameter optimization, the Taguchi method enables better control and understanding of the factors that affect product performance.

The design of the experiment was based on the Taguchi L9 orthogonal array, using three parameters with three levels each, as shown in Table 2. This design allowed for a total of only nine distinct experiments per material, in contrast to the full factorial experiment, which would have required 27 tests (3 to the power of 3).

**Figure 4:**
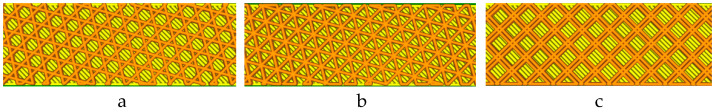
The profiles of the infill patterns: (**a**) tri-hexagonal, (**b**) triangular, and (**c**) octet.

The infill patterns of specimens are shown in Figure 4. Table 2 represents the parameters and their levels, and Table 3 shows the L9 orthogonal array. After the specimens were printed, they were weighed, and the density was measured by dividing their mass by their volume. It varied from 0.94 to 1.07 g/cm^3^ for nylon, and from 0.92 to 1.03 g/cm^3^ for ABS.

### 2.3. Experimental Procedure

The experimental procedure involved two main stages: 3D printing and tensile testing. The 3D printing was performed using Ultimaker S3 and Ultimaker S5. The tensile testing was conducted utilizing MTS Electromechanical Universal Test Systems, with a maximum rated force capacity of 25 kN.

The parameters for printing were modified using the Ultimaker Cura software, and an STL input file with a designated 3D part. Apart from the parameters that were examined, there were constant variables, that are summarized in Table 4.

After the printing process, the dimensions of a part were measured using a caliper, because they could be modified after being printed. Then, the FFF-printed parts were tested. The tensile testing was performed using an automated material-testing system, with crosshead speeds of 2 mm/s.

### 2.4. Data Interpretation and Optimization

The tensile test results were analyzed firstly by their means and delta values, to perform sensitivity analysis, and rank the parameters by significance. The Taguchi method offers the calculation of the signal-to-noise ratio number, to clearly see the effect of every one changing parameter on the experimental results, by reducing the effect of the other two. The larger-the-better condition was applied, as we wanted to investigate to what extent each parameter affected the results. This condition is defined with the application of the following Equation (1):

(1) $$S/N=-10\cdot log\left(\frac{1}{n}\sum_{i}^{n}\frac{1}{y_{i}^{2}}\right)$$

where n is the number of observations per test, and y is the observed data.

## 3. Results and Discussion

### 3.1. Experimental Results

The printed ABS and nylon parts, and the result of the tensile testing, are presented in this section. Overall, 18 samples were printed, with two repetitions of each of nine experimental runs, described in the DoE. Table 5 presents the ultimate tensile strength, yield strength, and Young’s modulus calculated. The test code was the parameter-level configuration corresponding to each experimental run, presented in Table 3. Additionally, the coefficients of variations of the determined parameters were calculated:

*k* = 100% *SD*/*x_m_* (2)

where *SD* is the standard deviation, and *x_m_* is the mean value. Thus, the *k* values of the ultimate tensile strength, yield strength, and Young’s modulus were equal to 4.7%, 3.9%, and 5.0% for ABS, and 2.0%, 3.3%, and 5.1% for nylon, respectively.

**Figure 5:**
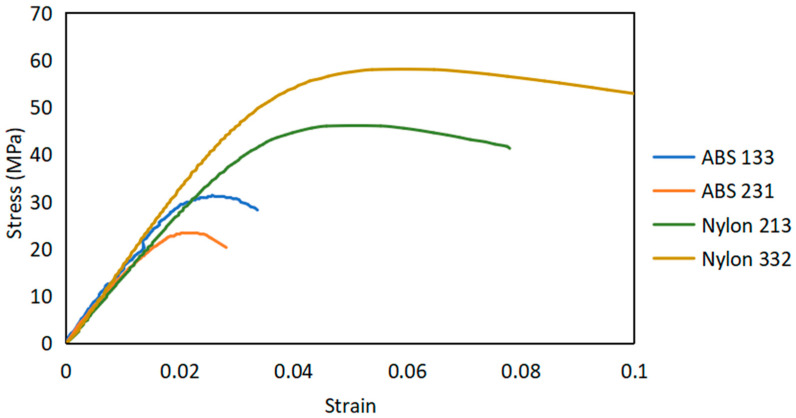
Stress–strain curves of ABS 133, ABS 231, nylon 213, and nylon 332 3D-printed parts.

**Figure 6:**
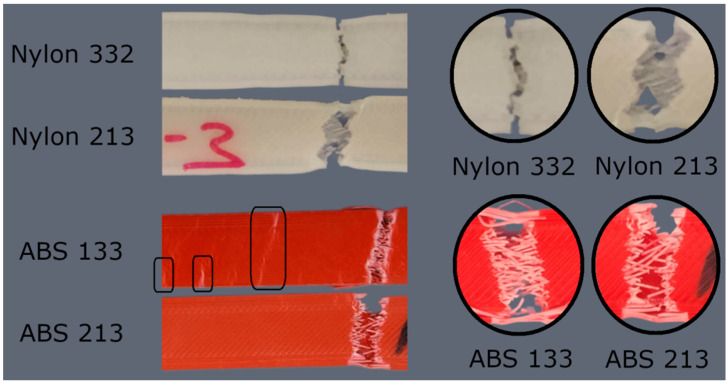
Tested 3D-printed specimens for ABS and nylon.

A graphical representation that illustrates the relationship between stress and strain for the weakest and strongest printed parts of nylon and ABS is shown in Figure 5. The 3D-printed specimens after tensile tests are presented in Figure 6. As one can see, nylon and ABS initially display linear elastic behavior, where stress and strain are directly proportional. As the load increases, the materials transition into the plastic-deformation region, and eventually reach their ultimate tensile strength.

At the point of failure, the nylon specimen undergoes necking (as depicted in Figure 6), which refers to a localized reduction in the cross-sectional area. This necking phenomenon arises due to the non-uniform stress–strain distribution within the material. As the material elongates, it becomes thinner at a specific location, resulting in a narrower region. The failure of nylon predominantly takes place at the necked region. The fracture occurs in a ductile manner.

During the tensile testing of ABS parts, it is common to observe the occurrence of multiple cracks (as indicated by the rectangular regions in Figure 6). Eventually, one of these cracks propagates, and leads to failure. ABS exhibits a combination of both ductile and brittle behavior. However, compared to nylon, the fracture of ABS occurs in a more brittle manner. ABS shows less plastic deformation before reaching the fracture point when compared to nylon, indicating a lower ability to sustain plastic deformation.

The experimental results are shown in Table 5. The ultimate tensile strength values for ABS and nylon exhibit variations, within the ranges of 23.3–31.3 MPa and 46.1–58 MPa, respectively. The yield-strength values range between 20.8–29 MPa for ABS, and 33.5–42.1 MPa for nylon. The Young’s modulus values range from 1.52–1.72 GPa for ABS, and 1.44–1.75 GPa for nylon.

Table 6 shows the calculated mean value of each result, consisting of the ultimate tensile strength, yield strength, and Young’s modulus, in terms of signal-to-noise (S⁄N) ratios. The delta is the difference between the corresponding category’s maximum and minimum mean values. According to this delta value, the ranking was made, to conclude the sensitivity analysis. 

**Figure 7:**
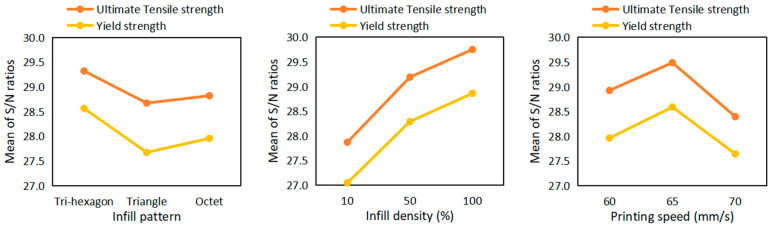

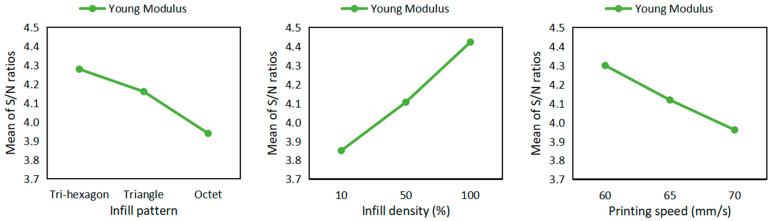
Sensitivity analysis for ABS parts in tensile testing.

The main plots of effects for ABS as a visual representation of the effect of each parameter on the results were depicted according to the mean value comparison, as shown in Figure 7.

According to our comparison and ranking, the infill density parameter had the greatest influence on the results, and the printing speed had the second highest. In contrast, the infill pattern of the specimen accompanied the least-sensitive effect. In the case of the infill pattern analysis, the ultimate tensile strength and yield strength had the highest larger-the-better results with the tri-hexagon design for ABS. The Young’s modulus was also noted to be higher with this structure. Unlike the tri-hexagon geometry, the triangular showed the weakest results for ultimate tensile strength and yield strength; and the octet, for Young’s modulus.

The infill pattern of ABS influences filament interaction inside the part. Therefore, the tri-hexagon structure resulted in higher mechanical properties. It enables more dense, crisscrossed supporting offsets that help layers to stick to each other and form a support. In the case of triangular and octet structures, they have fewer dense, crisscrossing offset numbers, and thus a weaker support, wherein octets showed slightly higher results than triangular structures.

Regarding the infill density for ABS material, the “higher infill density—better specimen characteristics” trend was observed. This is because the infill density is associated with the amount of material inside a part. Therefore, increasing the infill pattern causes an increase in the load-bearing capacity of a part, and a higher mechanical property. However, in this case, the sensitivity factor was more interesting than the ranking; that is, how far the results would be scattered along the value scale when the infill density was set to 10%, 50%, and 100%. It is important to note that the infill density percentage is not solely related to the extent to which the cross-sectional area will change as a result of printing. This is because various printing parameters remain constant, such as the wall thickness, number of bottom and top layers, nozzle diameter, printing orientation, and layer thickness, and the nozzle diameter ratio to the printing part’s XYZ dimensions.

The best printing speed for ABS material came up as being 65 mm/s. A higher or lower speed than 65 mm/s leads to a decrease in strength. The overall trend that could be observed for the results associated with printing speed was: at a lower printing speed, the interaction is stronger between the extruded layer and the solidified part. Thus, in most cases, the interaction becomes lower at higher speeds, and layers are loosely attracted to each other. In this work, such a trend was observed only for Young’s modulus, whereas one cannot distinguish a clear pattern or trends associated with printing speed for the ultimate tensile and yield strength. One possible reason for such results might be the small ranges selected for the printing speed [44]. It varied only from 60 to 70 mm/s. The tensile properties may be more sensitive for a larger speed range, which warrants further investigation.

**Figure 8:**
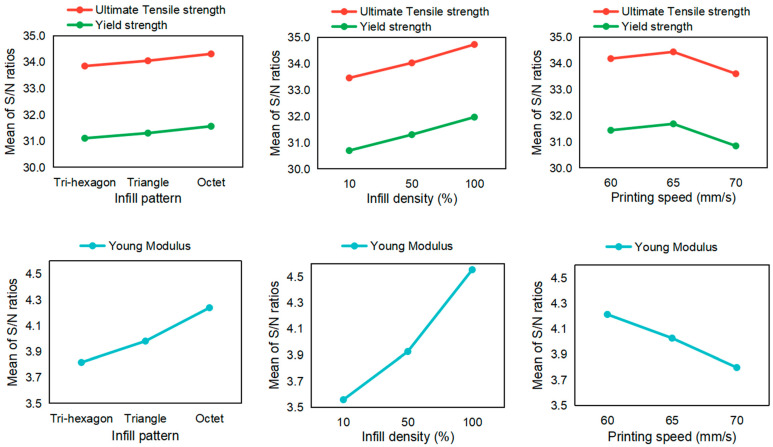
Sensitivity analysis for nylon parts in tensile testing.

A similar calculation methodology was also applied to the nylon material, to perform a sensitivity analysis of how different parameters affected the tensile testing results, as shown in Table 7. The results of the analysis are shown in Figure 8.

Next, in the same analysis for nylon material, the octet geometry resulted in the highest values for the material tensile and yield strength, and Young’s modulus. Moreover, 65 mm/s of printing speed was the optimal choice for nylon in terms of the tensile and yield strength values. Otherwise, nylon shows similar behavior patterns to ABS.

### 3.2. Validation of Results

After running the experiments and identifying the best parameters with the highest effect on the material performance under tensile tests, the hypothesis provided by the sensitivity analysis was checked. In order to do that, the new specimens were printed, following the best configuration parameters for each material. 

The specimens then underwent the corresponding test, and the results were calculated. As a result, the corresponding specimens resulted in the highest or the second-highest strength values. Obtaining the second-highest value might be related to the fact that the Taguchi method does not consider interaction effects in contrast to, for example, the full factorial DoE. The latter could give more precise results. The optimum parameter configurations for ABS and nylon parts are shown in Table 8.

## 4. Conclusions

A parametric study of the effect of printing parameters on the tensile mechanical properties of FFF-printed ABS and nylon is presented in this paper. Taguchi analysis was applied, to study the effect of the infill pattern, infill density, and printing speed on the ultimate tensile strength, yield strength, and Young’s modulus of the materials during tensile tests. The range of the parameter levels in the study was based on data available in the literature. 

During tensile testing, the infill density had the greatest influence on both ABS and nylon, as expected. The mechanical properties, including the ultimate tensile strength, yield strength, and Young’s modulus improved as the material infill density increased. These relationships were almost linear for all parameters, except for ABS’s ultimate tensile and yield strength. The slope was higher when the infill density changed from 10 to 50%, compared to when it changed from 50 to 100%. The printing-speed value of 65 mm/s was optimal for both materials. The infill pattern showed a limited effect on the tensile strength. ABS had a slightly higher load capacity with the tri-hexagon pattern, while nylon had a slightly higher load capacity with the octet structure.

Thus, an experimental investigation of the tensile features of 3D-printed ABS and nylon parts was conducted. The findings can serve as reference data for future parametric studies, and in the search for an optimal printing configuration for different materials. This work helps to fill the research gap associated with the difficulty in finding experimental data on a comparative tensile test of nylon and ABS.

## Figures and Tables

**Table 2:** Selected independent variables for the tensile test.

Parameters	Level 1	Level 2	Level 3
A: Infill pattern	Tri-hexagonal	Triangular	Octet
B: Infill density	10%	50%	100%
C: Printing speed	60 mm/s	65 mm/s	70 mm/s

**Table 3:** L9 Orthogonal array for the design of experiments (DOE).

#	A: Infill Pattern	B: Infill Density	C: Printing Speed
1	Tri-hexagonal	10%	60 mm/s
2	Tri-hexagonal	50%	65 mm/s
3	Tri-hexagonal	100%	70 mm/s
4	Triangular	10%	70 mm/s
5	Triangular	50%	65 mm/s
6	Triangular	100%	60 mm/s
7	Octet	10%	70 mm/s
8	Octet	50%	60 mm/s
9	Octet	100%	65 mm/s

**Table 4:** Constant variables for printing.

Parameter	ABS	Nylon
Layer height	0.15 mm	0.15 mm
Orientation	horizontal	horizontal
Wall thickness	1.3 mm	1.3 mm
Wall line Count	4	4
Horizontal expansion	0 mm	0 mm
Top/bottom thickness	1.2 mm	1.2 mm
Top layers	8	8
Bottom layers	8	8
Nozzle diameter	0.4 mm	0.4 mm
Fan speed	2%	40%
Printing temperature	240 °C	245 °C

**Table 5:** Calculations for each experimental test code in tensile testing.

Test Code	Ultimate Tensile Strength, MPa		Young’s Modulus, GPa		Yield Strength, MPa	
	ABS	Nylon	ABS	Nylon	ABS	Nylon
111	26.1	47.2	1.61	1.50	23.6	34.4
122	30.6	51.0	1.63	1.52	28.2	37.3
133	31.3	49.6	1.67	1.63	29.0	36.1
213	23.3	46.1	1.55	1.44	20.8	33.5
222	28.3	49.4	1.58	1.57	25.2	36.1
231	30.3	56.2	1.72	1.75	27.0	41.0
313	24.9	47.9	1.52	1.58	23.3	35.0
321	27.6	50.4	1.60	1.63	24.6	36.9
332	30.6	58.0	1.61	1.68	27.3	42.1

**Table 6:** The comparison of the mean values of the data, according to their categories and ranking of parameters, for ABS material.

S/N Ratios	Parameter Level	Infill Pattern	Infill Density	Printing Speed
Ultimate tensile strength	1	29.32	27.87	28.93
	2	28.67	29.19	29.49
	3	28.82	29.75	28.39
	Delta	0.65	1.88	1.09
	Rank	3	1	2
Young’s modulus	1	4.28	3.85	4.30
	2	4.16	4.11	4.12
	3	3.94	4.42	3.96
	Delta	0.34	0.57	0.34
	Rank	2	1	2
Yield strength	1	28.56	27.04	27.96
	2	27.67	28.29	28.59
	3	27.96	28.86	27.64
	Delta	0.89	1.82	0.95
	Rank	3	1	2

**Table 7:** The comparison of the mean values of the data according to their categories, and ranking of parameters for nylon material.

S/N Ratios	Parameter Level	Infill Pattern	Infill Density	Printing Speed
Ultimate tensile strength	1	33.85	33.45	34.17
	2	34.05	34.02	34.43
	3	34.31	34.72	33.60
	Delta	0.46	1.27	0.83
	Rank	3	1	2
Young’s modulus	1	3.82	3.56	4.21
	2	3.98	3.93	4.03
	3	4.24	4.55	3.80
	Delta	0.42	0.99	0.42
	Rank	2	1	2
Yield strength	1	31.10	30.70	31.44
	2	31.30	31.30	31.68
	3	31.56	31.97	30.84
	Delta	0.46	1.27	0.84
	Rank	3	1	2

**Table 8:** Best configuration parameter specimens, and the corresponding comparison with the set.

Material	Best Configuration Parameters			Comparison to Other Configuration Results
	Parameter 1	Parameter 2	Parameter 3	
ABS	Tri-hexagon geometric structure	100% infill density	65 mm/s printing speed	Highest
Nylon	Octet geometric structure	100% infill density	65 mm/s printing speed	The same, with the second-highest configuration

## Data Availability

The data presented in this study are available on request from the corresponding author.
